# Volume infusion cooling increases end-tidal carbon dioxide and results in faster and deeper cooling during intra-cardiopulmonary resuscitation hypothermia induction

**DOI:** 10.1186/s40635-015-0073-y

**Published:** 2015-12-29

**Authors:** Joshua W. Lampe, George Bratinov, Theodore R. Weiland, Uday Illindala, Robert A. Berg, Lance B. Becker

**Affiliations:** The Feinstein Institute for Medical Research, Northwell Health, 350 Community Drive, Manhasset, NY 11030 USA; Department of Anesthesiology and Critical Care, Children’s Hospital of Philadelphia, Philadelphia, PA USA; Department of Neonatology, Children’s Hospital of Philadelphia, Philadelphia, PA USA; ZOLL Circulation, Sunnyvale, CA USA

**Keywords:** Targeted temperature management, Therapeutic hypothermia, Intra-ischemia hypothermia, Cardiopulmonary resuscitation

## Abstract

**Background:**

Intra-arrest hypothermia induction may provide more benefit than inducing hypothermia after return of spontaneous circulation. However, little is understood about the interaction between patient physiology and hypothermia induction technology choice during ongoing chest compressions.

**Methods:**

After 10 min of untreated ventricular fibrillation, mechanical chest compressions were provided for 60 min (100 CPM, 1.25" deep) in 26 domestic swine (30.5 ± 1.7 kg) with concurrent hypothermia induction using one of eight cooling methods. Four cooling methods included volume infusion with cold saline or an ice particulate slurry through the femoral vein or carotid artery (volume infusion cooling group, VC); three included cooling via an intra-vascular heat exchange catheter, nasal cooling, or surface ice bags (no volume cooling group, NVC); and the other was a control group with no cooling (no cooling group, NC). Physiological monitoring included end-tidal carbon dioxide, aortic pressure, right atrial pressure, brain temperature, esophageal temperature, and rectal temperature.

**Results:**

During cardiopulmonary resuscitation (CPR), the volume infusion cooling group cooled faster and to lower temperatures than the other groups (VC vs. NVC or NC; ∆*T* = −5.6 vs. −2.1 °C or −0.6 °C; *p* < 0.01). The aortic pressure and right atrial pressure were higher in the volume cooling group than the other groups (VC vs. NVC or NC; AOP = 23.6 vs. 16.7 mmHg or 14.7 mmHg; *p* < 0.02). End-tidal carbon dioxide measurements during CPR were also higher in the volume cooling group (VC vs. NVC; EtCO_2_ = 23.4 vs. 13.1 mmHg; *p* < 0.05). Intra-corporeal temperature gradients larger than 3 °C were created by volume cooling during ongoing chest compressions.

**Conclusions:**

Volume infusion cooling significantly altered physiology relative to other cooling methods during ongoing chest compressions. Volume cooling led to faster cooling rates, lower temperatures, higher end-tidal carbon dioxide levels, and higher central vascular pressures.

IACUC protocol numbers: UPenn (803178), CHOP (997)

## Background

Cooling during ischemia seems to confer more benefit than cooling after reperfusion [1–3]. In animal models of myocardial ischemia, it has been shown that intra-ischemia hypothermia improves defibrillation success rate, [4] reduces the ratio of infarct area to area at risk in myocardial infarction, [5] and improves survival when emergency cardiopulmonary bypass is used to treat asphyxial cardiac arrest [6]. Clinical investigation also shows that intra-arrest cooling during emergency cardiopulmonary bypass treatment of refractory cardiac arrest results in improved neurological outcomes [7]. Due in part to this evidence, multiple cooling technologies have been tested in the field during cardiopulmonary resuscitation (CPR) in the treatment of out of hospital cardiac arrest [8–11].

We still do not understand many aspects of hypothermia induction during ongoing cardiac arrest. Hemodynamics are affected by a reduction in body temperature, [12, 13] particularly blood flow to the brain and the heart [14, 15]. Changes in blood flow are known to alter the dynamics of heat transfer in the body [16]. Cardiac arrest leads to the cessation of blood flow, and CPR is thought to restore 10–20 % of normal blood flow [17]. Therefore, hypothermia induction during cardiac arrest or cardiac arrest with ongoing CPR will be different than hypothermia induction with a beating heart.

Different cooling technologies are more dependent on blood flow for their effectiveness than others. Cooling technologies such as cooling blankets, intra-vascular heat exchange catheters, and nasopharyngeal cooling use heat exchange interfaces to withdraw heat from the body. These devices are highly dependent on heat convection by the blood to achieve cooling. Methods that use cold volume infusions rely on the energy balance of mixing a cold fluid with a warm one to reduce body temperature. Volume infusions allow for cooling to be supplied without heat convection by the blood, although temperature equalization in the body will be dependent on blood flow. Intravenous volume addition is going to increase the blood volume significantly and will change many aspects of the patient’s hemodynamics [18].

For all of these reasons, it seems likely that patients cooled intra-arrest with volume infusion methods may exhibit very different thermokinetic and physiological responses than patients cooled using heat extraction methods. In this manuscript, we report hemodynamic and thermokinetics measured in a highly instrumented swine model of prolonged CPR with concurrent hypothermia induction. Data are compared between groups that received volume infusion cooling (VC), no volume cooling (NVC), and no cooling (NC). This is a subgroup analysis from a larger study designed to study the impact of blood flow on thermokinetics during hypothermia induction.

## Methods

The study was approved by the Institutional Animal Care and Use Committee of the University of Pennsylvania (protocol # 803178) and the Children’s Hospital of Philadelphia (protocol # 997). All animals received treatment and care in compliance with the 1996 Guide for the Care and Use of Laboratory Animals by the National Research Council in accord with the USDA Animal Welfare Act, PHS Policy, and the American Association for Accreditation of Laboratory Animal Care. All experiments were conducted by qualified personnel.

### Animal preparation

Twenty-six domestic swine (30.5 ± 1.7 kg) were sedated with intramuscular ketamine (20 mg kg^−1^) and xylazine (2 mg kg^−1^), followed by induction of general anesthesia by mask administration of 4 % isoflurane in 100 % oxygen. After endotracheal intubation, a surgical plane of anesthesia was maintained with 1.5–2 % isoflurane and a mixture of air and oxygen, adjusted to achieve an inspiratory oxygen fraction of 0.4. The animals were mechanically ventilated with a pressure controlled ventilator (Modulus SE 7900; Datex-Ohmeda Inc., USA) with a tidal volume of 12 ml/kg, PEEP of 6 cm H_2_O, and rate of 12 breaths/min. The rate was titrated to maintain ETCO_2_ at 38–42 mmHg (NICO_2_; Novametrix Medical Systems Inc.).

To collect accurate brain temperature data as a function of anatomical location and time, seven temperature probes were placed in the brain. After aseptic preparation, seven thermocouples were advanced through three burr holes placed in the skull. A single thermocouple (IT-18) was advanced through a burr hole that was located on the left side of the intra-frontal suture. Triple thermocouple probes (IT-18(3)), spacing 0.5″ between thermocouples, were advanced through two burr holes drilled in the parietal bone, one on either side of the inter-parietal suture. Thermocouples were also placed in the esophagus and rectum (RET-1; PhysiTemp Instruments, Inc.).

Solid state pressure transducers (MPC-500; Millar Instruments) were advanced through introducers in the left femoral artery and vein to measure the aortic pressure (AOP) and the right atrial pressure (RAP), respectively. When required for hypothermia induction, an introducer was placed in the left femoral vein for femoral volume infusions or in the left femoral artery for carotid volume infusions. Carotid infusions were delivered through an interventional radiology catheter that was advanced from the femoral artery to the carotid artery under fluoroscopic guidance.

After surgical prep, low blood pressures, if present, were treated with a 20 ml/kg infusion of saline. Baseline measurements were recorded for 2 min, and then ventricular fibrillation (VF) was electrically induced. After 10 min of untreated VF, chest compressions were initiated at a rate of 100 per minute and a depth of 1.25 in. and cooling was initiated. Chest compressions were provided by a mechanical device developed specifically for use in swine models of cardiac arrest. After 60 min of chest compressions and cooling, the experiment was terminated.

### Cooling methods

Eight cooling technologies, both experimental and commercially available, were tested in this study. The target number of animals for each cooling technology (experimental group) was three. In some cases, the animal numbers are larger due to variability in the collected data or to accommodate technical failure in some aspect of an experiment. In addition, some data streams were excluded from analysis for technical reasons, such as signal loss during the experiment.

To investigate the impact of cooling technologies on CPR hemodynamics, the cooling technologies have been grouped into categories: volume infusion cooling (VC), no volume cooling (NVC), and no cooling (NC), as shown in Table 1. Hypothermia targets were established as follows: the Alsius IVTM system was set to target and maintain a body temperature of 32 °C and was run for 1 h; saline infusion cooling was set to deliver 3 l of ice cold saline at a rate of 4 l/h; slurry infusion cooling was set to deliver 3 l of ice particulate slurry at a rate of 5.2 l/h; the RhinoChill system evaporated 2 l of coolant at a gas flow rate of 40 l/min of O_2_ (it usually took about 45 min); large bags were filled with ice and water and placed on the abdomen, thorax, and neck for 1 h; and passive cooling was allowed to occur unimpeded for 1 h. Differences in the saline and slurry pump rates arose due to difficulties pumping the ice particulate saline at low flow rates.

**Table 1:** Map of experimental groups into cooling groups for analysis

Animal group	Volume cooling (VC)	No volume cooling (NVC)	No cooling (NC)
Femoral saline *n* = 3	X		
Femoral slurry *n* = 3	X		
Carotid saline *n* = 5	X		
Carotid slurry *n* = 3	X		
Alsius IVTM *n* = 3		X	
RhinoChill *n* = 3		X	
Ice bags *n* = 3		X	
Control *n* = 3			X

### Data analysis

Data were averaged over 67 1-min epochs (−2 ≤ *t* ≤ 65) and are presented in the figures as lines. For statistical comparison, data from each cooling group were compared and reported at 5-min intervals from minutes 15-65 and are presented in the figures as error bars. Data values were compiled and grouped using the scripting language Python (version 2.7). Statistical analyses were performed using R (version 3.2.1).

The experimental groups do not contain the same number of experiments (unbalanced experimental design). Therefore, we used the R library *lme4* to perform a linear fixed effects analysis of the relationships between the reported outcomes variables (e.g., EtCO_2_) and cooling group (e.g., VC). As fixed effects, we entered time and cooling group and their interaction. As random effects, we had intercepts for each animal. *p* values for the validity of the role of the cooling group on the outcome variable were obtained by likelihood ratio tests of the full model against a model that omitted the cooling group fixed effect. When the linear fixed effects analysis showed a significant contribution of the interaction between the time and cooling group, we performed a post hoc pairwise *t* test (with no *p* value adjustment) to determine which outcome variables were statistically different by cooling group as a function of time.

The solubility of carbon dioxide in the blood is temperature dependent. Before comparing the amount of CO_2_ delivered to the lungs during CPR, the EtCO_2_ measure had to be corrected for differences in temperature between the cooling groups. The temperature dependence of the partial pressure of carbon dioxide is described by the empirical equation [19]

1 $$ {P}_{{\mathrm{CO}}_{2_{{}_{\mathrm{hot}}}}}=\raisebox{1ex}{${P}_{C{O}_{2_{\mathrm{cold}}}}$}\!\left/ \!\raisebox{-1ex}{${10}^{0.019\varDelta T}$}\right. $$

where

2 $$ \varDelta T=T-{37}^{\circ}\mathrm{C}, $$

${P}_{{\mathrm{CO}}_{2_{{}_{\mathrm{hot}}}}}$is the partial pressure of CO_2_ in the blood stream at normal body temperature, ${P}_{{\mathrm{CO}}_{2_{{}_{\mathrm{cold}}}}}$ is the measured EtCO_2_ value, ∆*T* is the change in body temperature, and *T* is the experimental body temperature. For each epoch of data, the esophageal temperature was assumed to most closely correlate with the temperature of the blood in the lungs and was used in Eq. 2 to correct the measured EtCO_2_ values.

## Results

Table 2 shows the pre-arrest data for each of the animal groups as well as how many data streams were analyzed per group. There are no statistical differences in body weight or initial temperatures between the groups. There is a statistical difference in pre-arrest mean arterial blood pressures (MAP) and coronary perfusion pressures (CPP) in the volume cooling group when compared to the other groups. This difference was principally driven by low aortic blood pressure in the carotid infusion groups. This could be due to several factors such as the number and location of vascular introducers required for these experiments.

**Table 2:** Pre-arrest values for reported data

Experimental group	Mass	RecT	EsoT	BrainT	CPP	AOP	RAP	EtCO_2_
No cooling (NC)	30.1 ± 0.1 *n* = 3	36.3 ± 0.6 *n* = 3	35.8 ± 0.5 *n* = 3	35.3 ± 0.4 *n* = 3	84.2 ± 23.6 *n* = 3	98.4 ± 23.8 *n* = 3	14.3 ± 2.1 *n* = 3	38.4 ± 1.1 *n* = 3
No volume cooling (NVC)	30.4 ± 1.7 *n* = 9	36.7 ± 0.8 *n* = 5	36.3 ± 0.7 *n* = 9	36.0 ± 0.5 *n* = 9	79.9 ± 13.0 *n* = 9	93.5 ± 10.7 *n* = 9	13.5 ± 5.4 *n* = 9	39.6 ± 1.4 *n* = 9
Volume cooling (VC)	30.7 ± 1.0 *n* = 13	36.0 ± 2.6 *n* = 11	35.6 ± 2 *n* = 13	36.0 ± 1.0 *n* = 12	61.6 ± 22.3* *n* = 13	78.2 ± 20.6* *n* = 13	16.6 ± 4.9 *n* = 13	36.6 ± 8.3 *n* = 13

*Statistically significant difference in baseline parameters (*p* < 0.05)

Table 3 shows the results of the statistical modeling and likelihood ratio tests for the reported parameters. The intercept value represents the mean measure for all times in the VC group. The mean values may be adjusted up or down according to the effects of time and the cooling group as described in the rest of the fixed effects table. Our statistical question is if the cooling group had a significant effect on the measured parameters, and this is tested by the likelihood ratio test. For each measured parameter, the inclusion of the cooling group and the interaction between the cooling group and time led to a significant improvement in the model, as indicated by the likelihood ratio test *p* values, suggesting that the cooling group was an important factor in the design. As shown in the interaction column in Table 3, there was always a significant effect between the cooling group and time. Statistical differences between the cooling groups as a function of time were determined using post hoc pairwise *t* tests.

**Table 3:** Results of statistical modeling and likelihood tests

	Test of fixed effects				Likelihood ratio test		
Measurement	Intercept	Time	Cooling group	Interaction	Chi-square	DF	*p* value
MAP [mmHg]	*29.3*	−0.01 ± 0.04	*5.9 ± 1.3*	*−0.02 ± 0.002*	77.0	2	*<2.2E−16*
RAP [mmHg]	*18.3*	*0.1 ± 0.02*	*2.8 ± 0.8*	*−0.08 ± 0.008*	78.3	2	*<2.2E−16*
CPP [mmHg]	*7.2*	*−0.1 ± 0.05*	*5.5 ± 1.7*	*−0.1 ± 0.03*	24.5	2	*4.7E−6*
EtCO_2_ [mmHg]	*24.7*	*0.3 ± 0.04*	24.7 ± 3.5	*−0.2 ± 0.02*	80.0	2	*<2.2E−16*
BrainT [°C]	*34.6*	*−0.2 ± 0.003*	0.2 ± 0.2	*0.06 ± 0.002*	838.4	2	*<2.2E−16*
EsoT [°C]	*35.9*	*−0.2 ± 0.003*	0.2 ± 0.2	*0.06 ± 0.002*	838.4	2	*<2.2E−16*
RecT [°C]	*39.4*	*−0.1 ± 0.001*	*−1.5 ± 0.1*	*0.003 ± 0.0007*	1066.2	2	*<2.2E−16*

Statistically significant (*p* < 0.05) effects are presented in italicized text. Likelihood test shows that inclusion of cooling group and interactions between cooling group and time in the model significantly reduced the residual error relative to a model that depended only on time

**Fig. 1:**
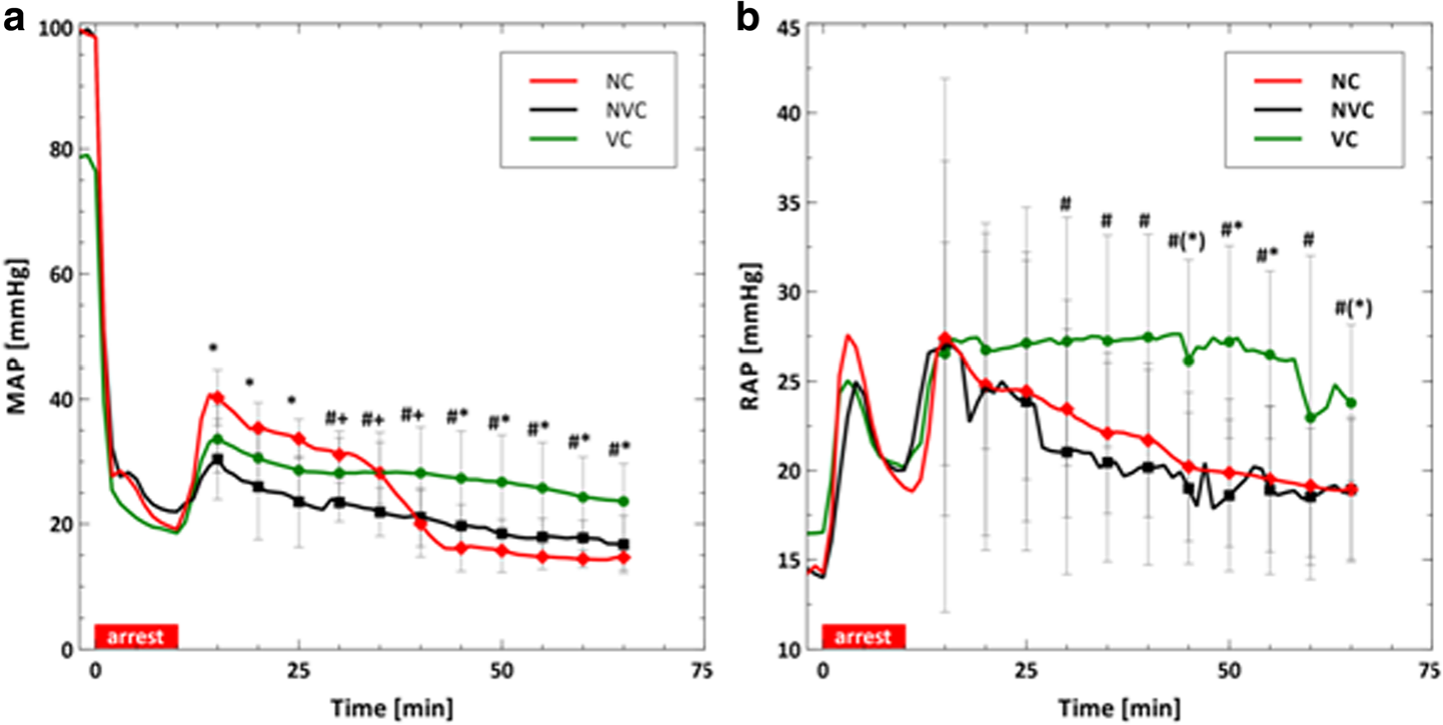
Average mean arterial (**a**) and right atrial (**b**) pressures reported every 5 min. The *asterisk symbol* [*] represents statistical difference (*p* < 0.05) between the VC and NC groups, and the *pound sign* [#] represents statistical difference between the VC and NVC groups. *Parentheses* around a symbol represent a trend (*p* < 0.1) with the symbols keeping the same meaning

The addition of fluid volume to the blood should raise blood pressures on the arterial and venous sides of the cardiovascular system. In these experiments, the addition of fluid volume was done continuously at relatively low flow rates: 67 ml/min for saline and 87 ml/min for slurry. Vascular pressures were monitored during treatment. Figure 1a shows the MAP as a function of time. Figure 1b shows the RAP over time. The addition of saline increased the MAP (*t* = 45 min) and the RAP (*t* = 45 min) in the VC group relative to the NVC and NC groups. The statistical differences in the RAP between the VC and the NC groups are not as consistent as observed in the MAP.

**Fig. 2:**
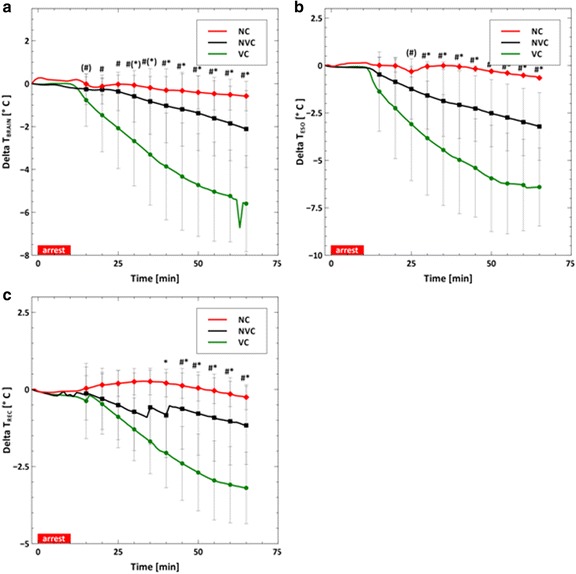
Average brain (**a**), esophageal (**b**), and rectal (**c**) temperatures reported every 5 min. The *asterisk symbol* [*] represents statistical difference (*p* < 0.05) between the VC and NC groups and the pound sign [#] represents statistical difference between the VC and NVC groups. *Parentheses* around a symbol represent a trend (*p* < 0.1) with the symbols keeping the same meaning

Figure 2 shows the temperature profiles of the brain (a), esophagus (b), and rectum (c) as a function of time. The VC cooling group had a significantly lower brain and esophageal temperature at *t* = 30 min and a significantly lower rectal temperature at *t* = 45 min. No significant differences between the NVC and NC groups were observed. After initiation of cooling, the esophagus was cooler than the rectum was, regardless of the cooling method. The coldest temperatures and fastest cooling rates were achieved with fluid volume infusion cooling.

**Fig. 3:**
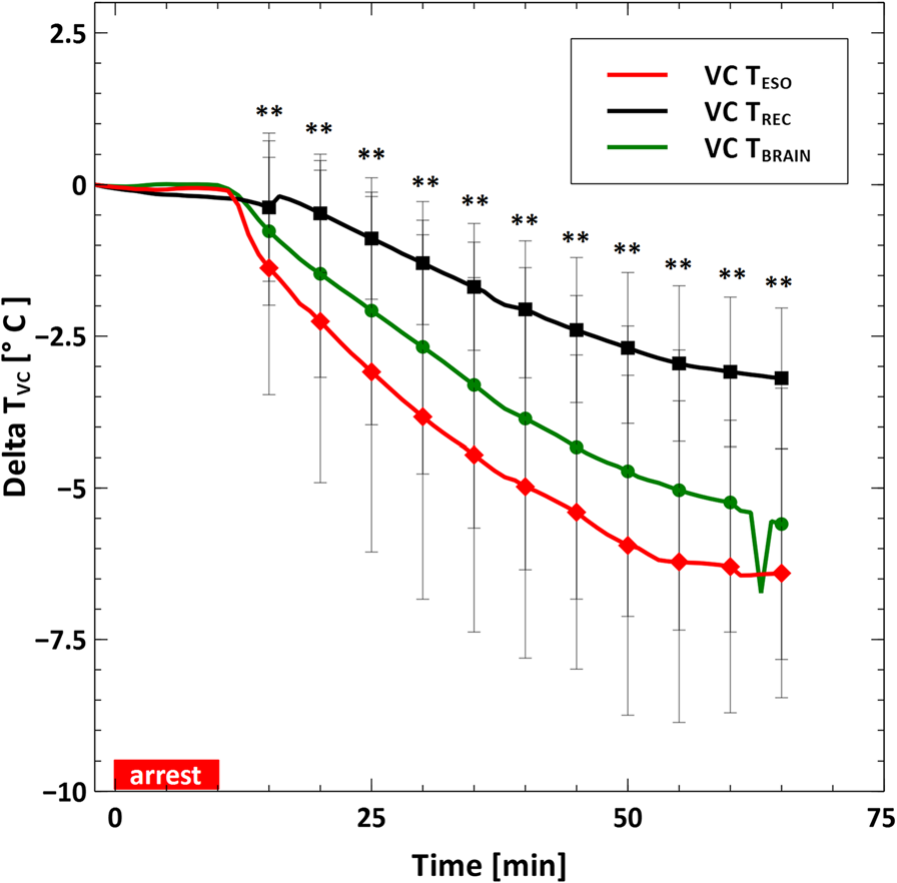
Comparison of body temperatures in the VC group. The *double asterisk* symbol (**) represents statistical difference between the rectal temperature and both the esophageal and brain temperatures. No differences were determined between the brain and esophageal temperatures

Figure 3 shows the temperature profiles of the brain, esophagus, and rectum in the VC cooling group. At all times, the brain and the esophageal temperatures were different than the rectal temperatures. However, statistical differences were not observed between the brain and the esophageal temperatures.

**Fig. 4:**
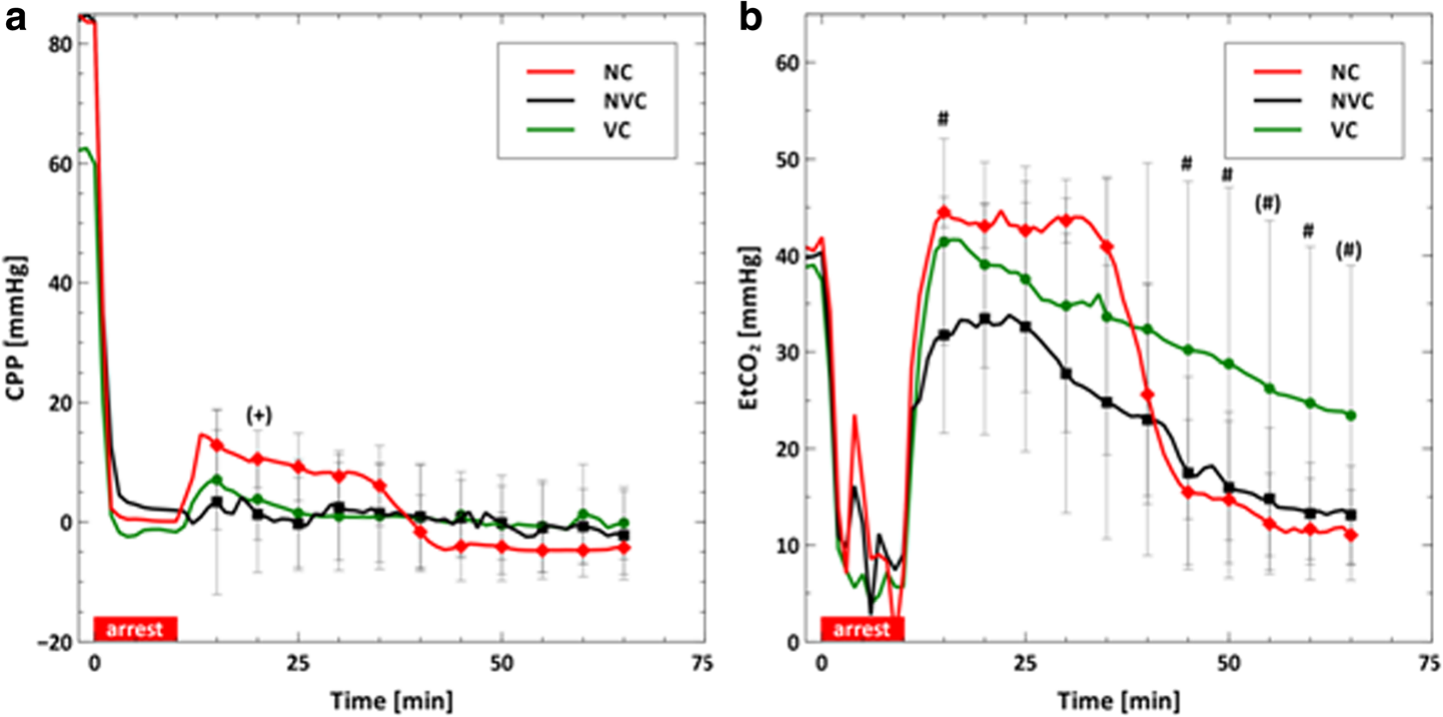
Average coronary perfusion pressure (**a**) and end-tidal carbon dioxide (**b**) reported every 5 min. The *asterisk symbol* [*] represents statistical difference (*p* < 0.05) between the VC and NC groups, and the *pound sign* [#] represents statistical difference between the VC and NVC groups. *Parentheses* around a symbol represent a trend (*p* < 0.1) with the symbols keeping the same meaning. The *plus sign* [+] represents a comparison of the NC and NVC groups

Figure 4 shows the CPP in a and the temperature corrected ETCO_2_ in b. The CPP did not differ among the cooling groups. ETCO_2_ measurements were higher in the VC group relative to the NVC group after *t* = 45 min of CPR. Given the variability of the ETCO_2_ measures during CPR and the small animal numbers in the NC group, no statistical differences were observed between the VC group and the NC group.

All CPR animals underwent necropsy after the experiment for visual and tactile assessment of the thoracic and abdominal cavities to assess potential trauma caused by 60 min of continuous chest compression. The animals that received a fluid volume infusion during CPR presented with significant fluid in the abdomen. The fluid in the abdomen did not include hemoglobin but did appear yellow, suggesting the presence of protein. Upon further inspection, it appeared that the fluid may have been released into the peritoneum through the spleen. The spleen was enlarged and covered with a white biofilm that was not present in the animals that did not receive fluid volume infusions. All animals sustained broken ribs (5.3 ± 1.2 ribs/animal). Twenty-one out of 25 animals exhibited a cramped left ventricle (stone heart), as observed by visual and tactile assessment. The right ventricle showed evidence of distension (loss of muscle tone) in 25 of 25 animals, as observed by visual and tactile assessment. Atelectasis was always observed in the lung tissue positioned under the heart. Blebbing was also observed on the surface of the lung. No damage to the major blood vessels was observed.

## Discussion

Our data demonstrates that cooling technology choice can lead to significantly different hemodynamics and thermokinetics during intra-CPR cooling. As shown in Figs. 1 and 4, volume infusion cooling changes the central blood pressures and increases EtCO_2_, a surrogate for cardiac output, [20, 21] during prolonged CPR. The data shown in Fig. 2 suggests that volume infusion cooling was more effective, e.g., faster cooling and colder temperatures, during intra-CPR cooling. These differences take relatively long times (5–30 min after initiation of cooling) to manifest, suggesting that they become more important as resuscitation efforts are prolonged. Body temperature gradients were created by inducing therapeutic hypothermia without a normal heart rhythm. These data are important because they add to our understanding of the impact of the cooling method on intra-ischemia therapeutic hypothermia, which has been shown to be protective in a variety of species and injury models [1–3].

### Effect of volume infusion on blood pressures and quality of CPR

The addition of volume before the initiation of chest compressions has been reported to increase the RAP, thereby decreasing the CPP [5, 18]. As shown in Fig. 1, the slow addition of volume increases both the AOP and RAP and is therefore CPP neutral, shown in Fig. 4. One possible explanation for the different result in this study is that saline or slurry was infused at a rate less than 90 ml/min, while previous publications used a rate of 140 ml/min [5] or as quickly as possible [18].

Interestingly, the slow addition of fluid volume increased EtCO_2_ as shown in Fig. 4. EtCO_2_ is thought to be a surrogate for CC quality, [20, 21] correlating with venous delivery of blood to the heart. We posit that EtCO_2_ was improved in the VC group because the imposition of a physiologically forward flow via fluid infusion, either arterial or venous, had the effect of increasing venous return of blood to the heart.

In combination, these data suggest that slow volume infusion has a net positive impact on blood flow generated by chest compressions.

### Effect of volume infusion on temperatures

Comparing the rectal, brain, and esophageal temperatures within the VC cooling group, shown in Fig. 3, it is apparent that intra-CPR cooling can create temperature gradients within the body. In the VC group, the maximum temperature difference between the rectal and esophageal temperatures is 3 °C and occurs at the end of the experiment. The maximum temperature difference between the esophageal and brain temperatures is 2 °C, which occurs at 15 min. These temperature differences are approximately the same size as the target temperature reduction and are consistent with previously published data [22, 23].

The VC group cools faster and deeper than the NVC group or the NC group, as shown in Fig. 2. The addition of fluid volume should increase convective heat transfer during CPR. The NVC methods rapidly cooled the tissue volume closest to the heat exchange interface (data not shown) but were dependent on CPR-generated blood flow for convective heat transfer. Fluid volume infusion in the VC cooling group increases convective heat transfer relative to the other groups. The pump flow rates were small relative to the typical cardiac output for a 30-kg swine, ~67 ml/min for saline and ~87 ml/min for ice particulate saline. However, CPR generates relatively low volumetric blood flow, ~ 500–1000 ml/min. Therefore, the pumps increase the net forward blood flow during CPR by approximately 20 %. This percentage may increase with time as CPR efficacy declines. Improved convective heat transfer conferred a significant heat transfer advantage to volume infusion cooling during low blood flow states.

### Study limitations

This study was performed on adolescent swine. While there are clear physiological differences between the animal subjects used in this study and the typical cardiac arrest patient, the chosen size/weight and species are most commonly used in pre-clinical cardiac arrest/CPR studies. The animals in this study were never resuscitated. As a result, the effects of hypothermia technology choice on return of spontaneous circulation and survival remain unknown. Coronary perfusion pressures were not very high, suggesting that CPR was not optimal. While it would be expected that convective heat transfer would increase with improved CPR, this is unlikely to change the conclusions of the study because CPR results in less blood flow than a beating heart and more blood flow than untreated cardiac arrest, regardless of its efficacy.

Subgroup analyses by cooling methods shown in Table 1, e.g., femoral saline, are not reported because the analysis did not demonstrate any significant differences between subgroups for any measured parameter. The data that are reported here are highly variable between animals, particularly animals undergoing prolonged chest compression. As a result, the small number of animals per group (three in most cases) was not sufficient to provide statistical findings.

## Conclusions

Volume cooling during CPR resulted in faster cooling and achieved lower final temperatures compared with no volume cooling and no cooling. In addition, volume cooling was associated with higher end-tidal carbon dioxide, suggesting increased cardiac output. Temperature gradients between 2 and 3 °C are created in the body during intra-CPR cooling.
